# Intravenous thrombolysis before endovascular therapy for acute ischemic stroke due to tandem lesions: a systematic review and meta-analysis

**DOI:** 10.1007/s10143-025-03786-6

**Published:** 2025-09-08

**Authors:** Aaron Rodriguez-Calienes, Martha I. Vilca-Salas, Jason Z. Gao, Jenny K. Huynh, Arsh Manazir, Anish K. Venkatesan, Yujing Lu, Venkat Uppalapti, Cristian Morán-Mariños, Mohamed Elfil, Amer M. Malik, Dileep R. Yavagal, Santiago Ortega-Gutierrez

**Affiliations:** 1 Department of Neurology, Jackson Memorial Hospital, University of Miami Miller School of Medicine, Miami, FL USA; 2 Facultad de Medicina Alberto Hurtado, Universidad Peruana Cayetano Heredia, Lima, Peru; 3 Department of Biostatistics, University of Iowa, Iowa, IA USA; 4 Vicerrectorado de Investigación, Unidad de Investigación en Bibliometría, Universidad San Ignacio de Loyola, Lima, Peru; 5 Department of Neurology and Neurosurgery, Jackson Memorial Hospital, University of Miami Miller School of Medicine, Miami, FL USA; 6 Department of Neurology, Radiology & Neurosurgery, University of Iowa Hospitals and Clinics, Iowa, IA USA

**Keywords:** Tandem stroke, Endovascular thrombectomy, Stroke, Intravenous thrombolysis, Intracranial hemorrhage

## Abstract

The role of intravenous thrombolysis (IVT) in patients with tandem lesions (TL) undergoing endovascular thrombectomy (EVT) for acute ischemic stroke (AIS) remains a subject of ongoing debate. The substantial clot burden and the potential need for periprocedural antiplatelet therapy during emergent carotid stenting (CAS) add to the complexity of treatment decisions. This study aims to systematically review and meta-analyze the literature to evaluate the comparative safety and efficacy of IVT plus EVT versus EVT alone in AIS patients with TL. A systematic search was conducted across four databases to identify studies comparing outcomes for patients with TL receiving IVT prior to EVT with those receiving EVT alone. Outcomes of interest included symptomatic intracranial hemorrhage (sICH), functional independence (90-day modified Rankin Scale 0–2), successful reperfusion (modified Thrombolysis in Cerebral Infarction 2b-3), and 90-day mortality. We performed a random-effects meta-analysis to calculate pooled odds ratios (OR) for each outcome and conducted a sensitivity analysis for patients who underwent emergent CAS. The analysis included 24 studies with a total of 3,294 patients: 1,860 (56.5%) received IVT plus EVT, and 1,434 (43.5%) received EVT alone. There was no significant increase in the risk of sICH between the IVT + EVT and EVT alone groups (IVT + EVT: 8.4% vs. EVT alone: 8.8%; OR = 0.90; 95%CI 0.67–1.21; I^2^ = 5%). The sensitivity analysis showed no significant difference in sICH between patients treated with IVT before emergent CAS and those who received CAS alone (IVT + CAS: 10.6% vs. CAS alone: 10.9%; OR = 0.78; 95%CI 0.44–1.37; I^2^ = 0%). Patients treated with IVT before EVT had significantly higher odds of functional independence (IVT + EVT: 52.6% vs. EVT alone: 44.1%; OR = 1.34; 95%CI 1.13–1.59; I^2^ = 2.5%) and successful reperfusion (IVT + EVT: 83.3% vs. EVT alone: 79.8%; OR = 1.47; 95%CI 1.14–1.89; I^2^ = 14%). Additionally, the IVT + EVT group had lower odds of 90-day mortality (IVT + EVT: 13.4% vs. EVT alone: 21.1%; OR = 0.61; 95%CI 0.47–0.78; I^2^ = 0%). In conclusion, the current meta-analysis provides supporting evidence indicating that the combination of IVT and EVT does not increase the risk of sICH in patients with TL, even in the context of emergent CAS. Notably, IVT + EVT may offer potential benefits in achieving functional independence, successful reperfusion, and reducing mortality compared to EVT alone. However, randomized controlled trials are needed to validate these findings.

**Supplementary Information:**

The online version contains supplementary material available at 10.1007/s10143-025-03786-6.

## Introduction

Tandem lesions (TL) constitute a singular entity defined by the stenosis or occlusion of an extracranial segment of the internal carotid artery (ICA) alongside an ipsilateral intracranial large vessel occlusion (LVO), accounting for about 10–20% of acute ischemic strokes (AIS) [1–3]. While the benefit of endovascular treatment (EVT) and intravenous thrombolysis (IVT) is well-established in LVO-AIS, the management of TL in the acute phase presents unique challenges. Observational studies suggest that emergent carotid artery stenting (CAS) may improve clinical outcomes and reperfusion rates [4, 5]. Current randomized controlled trials (RCTs), including TITAN (Thrombectomy in Tandem Lesions) [6] and EASI-TOC (Endovascular Acute Stroke Intervention-Tandem Occlusion) [7]are actively investigating the optimal endovascular approach for treating TL.

The role of IVT before EVT in TL remains controversial. Patients with TL often experience more severe perfusion deficits, and the concurrent use of antiplatelet therapy with IVT in those undergoing emergent CAS may elevate the risk of hemorrhagic complications [8]. The Improving Reperfusion Strategies in Acute Ischemic Stroke (IRIS) meta-analysis [9]which pooled patient-level data from six RCTs comparing IVT + EVT versus EVT alone, found no significant treatment effect interaction on functional outcomes in TL. However, this analysis did not specifically assess the treatment effect of the different endovascular approaches for TL (CAS with or without angioplasty vs. angioplasty alone), nor did it examine the risk of hemorrhagic complications, leaving critical questions unanswered.

There have been no RCTs comparing IVT + EVT to EVT alone for TL; thus, current evidence relies predominantly on observational, non-randomized cohort studies [10, 11]. While some meta-analyses have attempted to synthesize the available literature, they have been constrained by small sample sizes and limited search strategies, often excluding the potential impact of emergent CAS on IVT-related safety outcomes. As research comparing IVT + EVT and EVT alone in TL has grown, a comprehensive synthesis is needed. Therefore, we aimed to conduct an extensive systematic review and meta-analysis to assess the comparative safety and efficacy of IVT + EVT versus EVT alone for AIS due to TL. Furthermore, we evaluated whether the combination of IVT with emergent CAS yields superior outcomes compared to emergent CAS alone.

## Materials and methods

### Protocol and guidance

This systematic review and meta-analysis were conducted in accordance with the Preferred Reporting Items for Systematic Reviews and Meta-Analyses (PRISMA) reporting guideline [12]. The study protocol and methods were registered with PROSPERO a priori (CRD42024520443).

### Search strategy and eligibility criteria

A systematic literature search was conducted by a medical librarian (C.M.M) across multiple databases including the Ovid Embase, PubMed, Scopus, and Web of Science. The search aimed to identify relevant articles published from the earliest records available in each database up to February 2024. Search algorithms were tailored to the specifications of each database, incorporating keywords such as “tandem*” or “thrombolysis”. Further details of the complete search strategy are provided in Supplementary Table 1.

Included studies utilized a randomized controlled trial or non-randomized design (including open-label trials, real-world or cohort observational studies, and case series) that compared the safety and effectiveness of IVT plus EVT versus EVT alone and were conducted in adult patients (≥ 18 years old) with anterior circulation AIS due to TL. Studies were excluded if they were review articles, abstracts, editorials, letters, animal studies, or case reports. Supplementary Table 2 provides a more detailed representation of the inclusion and exclusion criteria.

### Study selection and data collection process

The search strategy was systematically applied to each database. Retrieved records were managed using EndNote X9 software, where duplicate entries were identified and removed. Subsequently, the records were exported in.XML format from EndNote X9 to Mendeley for further organization. Mendeley facilitated the conversion of data into the RIS format, enabling utilization of the Rayyan tool (https://www.rayyan.ai/) for expert revision. Duplicate articles were meticulously eliminated, and four independent reviewers (JG, JKH, AM, and AKV) conducted the initial screening of titles and abstracts, adhering strictly to the predefined inclusion criteria to identify potentially relevant articles. Any discrepancies between reviewers were resolved through initial discussion, and in cases of persistent disagreement, a third reviewer (NMCH) acted as an arbitrator to reach consensus.

The extracted data included the following information: year of publication, study design, study period, patient characteristics (age, female participants), clinical characteristics (baseline National Institute of Health Stroke Scale [NIHSS], baseline Alberta Stroke Program Early Computed Tomography Score [ASPECTS]), IVT treatment protocol, and the prioritized outcomes.

### Outcomes and prioritization

The primary outcome was determined as the incidence of symptomatic intracranial hemorrhage (sICH), as defined by each study. The secondary outcome was determined as the proportion of functional independence at 90 days, defined by a modified Rankin Scale (mRS) score of 0–2. The mRS is an ordinal scale (0, no symptoms; 6, death) that reflects the degree of disability after a stroke. A score of 0 to 2 reflects independence for activities of daily living [13]. 

Additional outcomes included excellent clinical outcome (90-day mRS score of 0–1), successful reperfusion at final digital subtraction angiogram, defined as a modified Thrombolysis in Cerebral Infarction (mTICI) scale score 2b-3, complete reperfusion (mTICI scale score of 3), and all-cause mortality by 90 days.

### Risk of bias in individual studies

The quality of the studies included in this analysis was evaluated based on the guidelines outlined in the Cochrane reviewers’ handbook [14]. For the methodological assessment of non-randomized studies, two independent reviewers (ARC and MIVS) utilized the Risk of Bias in Non-randomized Studies of Interventions (ROBINS-I) tool [15]. Publication bias was assessed using Egger’s test and visual inspection of the funnel plots for asymmetry for each outcome of interest [16]. 

### Data synthesis

A meta-analysis was conducted when there were at least three studies reporting the same effect estimate for a specific outcome. In primary analyses, we calculated the odds ratios (ORs) and their corresponding 95% confidence intervals (CIs) for each outcome of interest using random-effects models with the Mantel-Haenszel method. Subgroup analyses were conducted based on study design (prospective vs. retrospective). A sensitivity analysis was restricted to studies comparing IVT + CAS versus CAS alone.

Mixed-effects logistic meta-regression models were used to assess the effects of selected variables in explaining study-level heterogeneity, as described in the “metafor” documentation [17]. Two models were used to study the associations between IVT + EVT and functional independence with moderators age or NIHSS. For studies without available mean values of age or NIHSS, medians were used as estimates of mean, as those studies have sample sizes greater than 25 [18]. 

We utilized the Cochran Q and I [2] tests to evaluate statistical heterogeneity. An I [2] statistic with values of > 25%, > 50%, and > 75% indicated low, considerable, and substantial levels of heterogeneity, respectively [19]. All statistical tests were 2 sided with a significance threshold of *p* ≤ 0.05. All analyses and plots were generated using R statistical software (version 4.3.1) and R Studio.

## Results

### Study selection and characteristics

A total of 8,522 titles and abstracts were initially identified, with 414 proceeding to full-text evaluation after screening. Ultimately, 24 studies met the eligibility criteria (Supplementary Fig. 1) and were included in the analysis, representing data from 19 countries. Baseline characteristics for each study are provided in Table 1.

**Table 1:** Characteristics of included studies

Study	Study design	Country	Study period	No. of patients	Intervention No.		Age, y	Female (%)	Baseline NIHSS	ASPECTS	Time (OTG), min	IVT protocol
					IVT + EVT	EVT						
Mujanovic et al., 2024 [20]	Secondary analysis of trial	Switzerland, Finland, France, Germany, Spain, Austria, UK, Canada	2017–2021	63	33	30	69 [61–76]	17 (27)	17 [14–19]	8 [7–9]	-	IV t-PA (0.9 mg/kg bodyweight with a maximum dose of 90 mg per patient) was administered for 60 min with 10% of the calculated dose given as an initial bolus.
Shah et al., 2023 [21]	R, Multicenter study	USA	2012–2019	78	45	33	-	-	-	-	-	t-PA, dose not specified.
Rodriguez-Calienes et al., 2023 [10]	R, Multicenter registry	USA, Spain	2015–2020	512	218	294	68 [59–76]	154 (30)	16 [11–20]	8 [7–9]	375 [217–706]	At the discretion of the treating clinician.
Enriquez et al., 2023 [22]	R, Single center study	Norway	2017–2020	90	51	39	68 [56–74]	29 (32)	15 [10–19]	-	248 [181–340]	t-PA, dose not specified.
Havlicek et al., 2023 [23]	R, Multicenter study	Czech Republic, Slovakia	2016–2022	300	224	76	67.3 ± 10.2	94 (31)	17	-	-	NS
Ingleton et al., 2023 [24]	P, Single center study	UK	2010–2020	102	76	26	67 [57–72]	32 (31)	18 [13–23]	-	269 [195–343]	IV t-PA 0.9 mg/kg body weight up to 6 h from onset for anterior circulation infarcts and up to 12 h for posterior circulation infarct.
Sanak et al., 2022 [25]	P, Multicenter study	Czech Republic	2019	194	131	63	68.7 ± 11.5	78 (40)	15	-	-	NS
Cirio et al., 2021 [26]	R, Single center study	Argentina	2015–2019	40	22	18	65 ± 13.81	13 (33)	13.5 [9–18]	6 [5–8]	298 [220–582]	t-PA, dose not specified.
Anadani et al., 2021 [11]	R, Multicenter registry	USA, France, Italy, Germany, Greece, Spain, Switzerland	2012–2017	602	380	222	62.9	194 (32)	16	-	-	NS
Bracco et al., 2021 [27]	R, Multicenter study	Italy	2015–2019	227	146	81	65.9 ± 12.9	73 (32)	16 [11–20]	9 [8–10]	253.6 ± 118.4	NS
Da Ros et al., 2020 [28]	R, Multicenter study	Italy	2018–2019	95	45	50	64.6 ± 12.6	26 (37)	16.8 ± 5.5	8	284.5 ± 156.4	NS
Fernandez-Menendez et al., 2020 [29]	P, Single center study	Spain	2011–2018	99	21	78	67.5 ± 9.5	22 (22)	15 [13–20]	8 [8–9]	207.12 ± 88.1	NS
Sojka et al., 2020 [3]	P, Single center study	Poland	2016–2019	34	23	11	70.6	10 (29)	16.4 ± 5.4	-	-	NS
Pikija et al., 2019 [30]	R, Multicenter study	Austria, Slovenia	2012–2016	53	37	16	76 [65–83]	25 (47)	20 [16–23]	8 [7–9]	-	t-PA, dose not specified.
Park et al., 2019 [31]	R, Single center study	Korea	2011–2017	42	29	13	70.9 ± 8.8	8 (19)	13.2 ± 5.9	-	-	Patients presenting within 4.5 h of symptom onset and without contraindications to IVT were treated immediately with t-PA (0.9 mgkg^−1^).
Bücke et al., 2018 [32]	R, Single center study	Germany	2010–2017	222	53	169	67.8 ± 12.8	71 (32)	14.8 ± 8.1	-	216 ± 66	NS
Eker et al., 2018 [33]	R, Multicenter study	Switzerland, France	2010–2015	121	63	58	72 [61–79]	31 (26)	17 [12–20]	-	262 [194–356]	IV t-PA 0.9 mg/kg, 10% of the dose as bolus and remainder over 60 min, administered to patients within a maximum of 4.5 h after stroke onset.
Sallustio et al., 2017 [34]	P, Single center study	Italy	2009–2016	72	39	33	65.6 ± 12.8	28 (39)	19 ± 2.9	6.9 ± 2.28	225	IVT was administered (t-PA 0.9 mg/kg; 10% of the dose as a bolus and the remaining infused over 60 min) within 4.5 h after symptom onset and continued during the endovascular procedure.
Fahed et al., 2016 [35]	R, Single center study	France	2011–2014	70	52	18	62.6 ± 11.3	21 (30)	16 [11–20]	7 [5–8]	272.3 ± 83.0	IV t-PA (0.9 mg/kg) or tenecteplase (dose not specified).
de Lucena et al., 2015 [36]	R, Single center study	Brazil	2009–2014	20	12	8	67.1 ± 11.4	6 (30)	16 [13-21.5]	9	-	t-PA, dose not specified.
Lockau et al., 2015 [37]	R, Single center study	Germany	2010–2013	37	20	17	63	10 (27)	17	-	-	Patients within 4.5 h from symptom onset and no contraindications received IV thrombolysis with t-PA at a maximum dose of 0.9 mg/kg.
Behme et al., 2015 [38]	R, Multicenter study	Germany	2007–2014	170	122	48	64	51 (30)	15	7	-	NS
Puri et al., 2015 [39]	R, Multicenter study	USA, Korea	2006–2013	28	11	17	58.7	9 (32)	18.5 ± 5.9	-	-	IV t-PA, dose not specified.
Heck et al., 2014 [40]	R, Single center study	USA	2011–2013	23	7	16	69.5	-	17.8 ± 4.3	7.7 ± 1.22	-	t-PA, dose not specified.

*IVT *Intravenous thrombolysis, *EVT *Endovascular therapy, *R *Retrospective, *P *Prospective, *t-PA *tissue Plasminogen activator, *OTG *onset to groin

The pooled analysis included 3,294 patients with TL, of whom 1,860 (56.5%) underwent IVT + EVT and 1,434 (43.5%) underwent EVT alone. Across all patients, 31.2% were female and 68.8% were male, with a mean age ranging from 58 to 71 years. Median baseline NIHSS scores ranged from 13.5 to 20, and ASPECTS values ranged from 6 to 9. The median symptom onset-to-groin (OTG) time varied from 225 to 375 min.

### Risk of bias

The overall risk of bias across all studies was rated as serious, as detailed in Supplementary Fig. 2. Three studies [10, 11, 20] reported multivariable adjusted outcomes to help control for potential confounding factors. However, the remaining studies, due to their retrospective design, received a serious rating for bias in participant selection. All studies were rated as having a serious risk of bias in the classification of the intervention, and we lacked sufficient information to assess bias related to treatment deviations. Seven studies were rated as having a serious risk of bias due to missing data, as they excluded participants from the final analysis at some point during the study. All studies had a critical risk of bias in outcome measurement due to the absence of blinding. Additionally, they were classified as having a moderate risk of bias in reporting results, as there was uncertainty regarding unspecified outcomes or subgroup analyses. In the overall rating, only three studies were rated as moderate, while the rest were deemed to have a serious risk of bias.

Visual inspection of the funnel plots revealed asymmetry for functional independence, excellent clinical outcome, complete reperfusion, and 90-day mortality, suggesting potential publication bias, while the remaining funnel plots appeared symmetrical (Supplementary Fig. 3). Egger’s test for our priotized outcome showed significant publication bias for 90-day mortality (Supplementary Table 3).

### Symptomatic intracranial hemorrhage

**Fig. 1:**
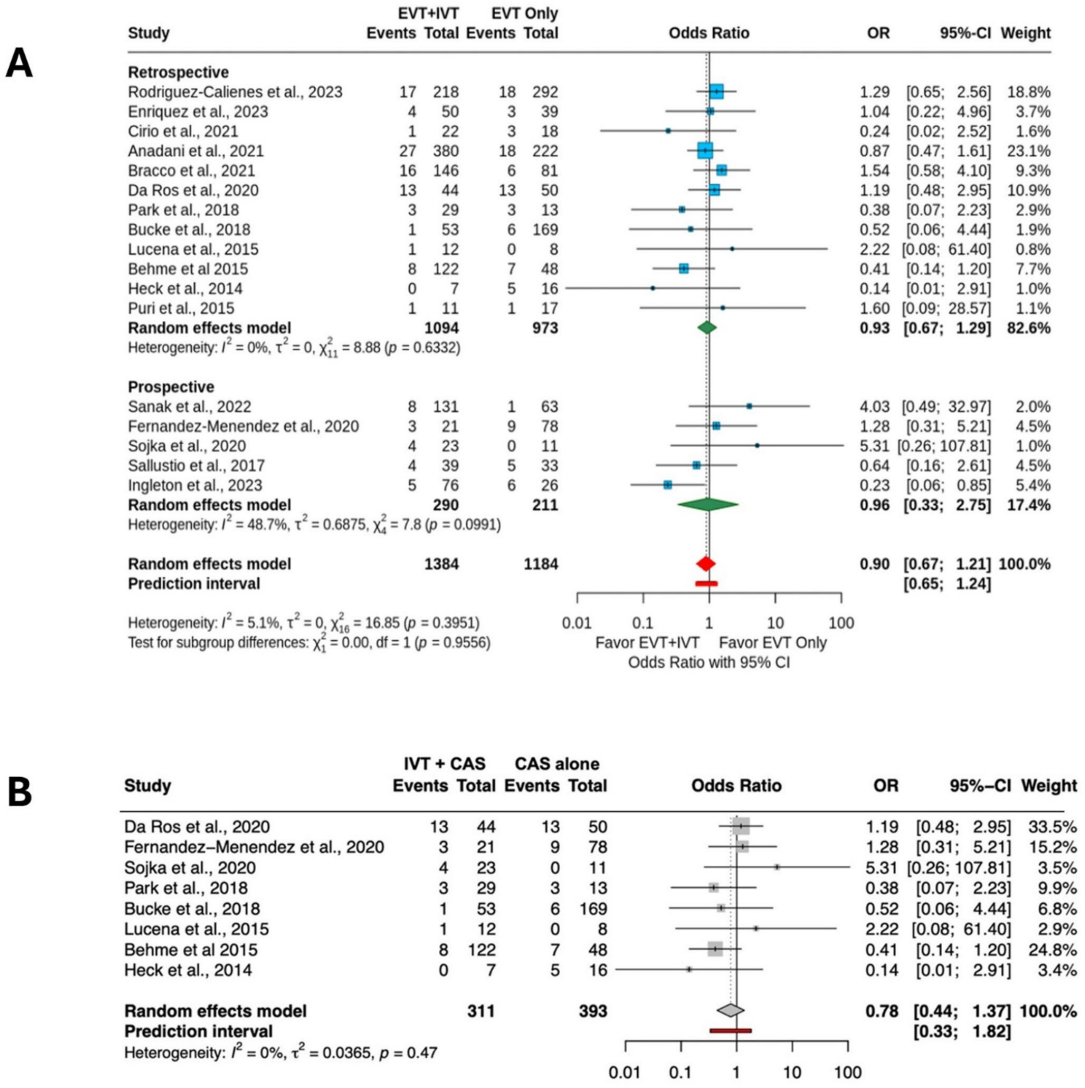
**A** Meta-analysis of symptomatic intracranial hemorrhage in studies comparing intravenous thrombolysis plus endovascular thrombectomy (IVT + EVT) vs. endovascular thrombectomy (EVT) alone. **B** Sensitivity analysis of studies comparing IVT plus emergent carotid artery stenting (IVT + CAS) vs. CAS alone

Rates of sICH were reported in 17 studies (2,568 patients). The odds of sICH did not significantly differ between IVT + EVT and EVT alone (IVT + EVT: 8.4% vs. EVT alone: 8.8%; OR = 0.90; 95% CI 0.67–1.21), with low heterogeneity (I^2^ = 5.1%). Numerically, the odds were slightly higher in prospective studies (OR = 0.96; 95% CI 0.33–2.75) compared to retrospective studies (OR = 0.93; 95% CI 0.67–1.29), although the CIs overlapped (Fig. 1A). Meta-regression found no significant influence of baseline variables (age: *P* = 0.75, I² = 7.06%; NIHSS: *P* = 0.86, I² = 4.22%) on the association between IVT + EVT and sICH.

Eight studies (704 patients) assessed the risk of sICH between IVT + CAS vs. CAS alone. The pooled analysis showed no significant difference in sICH rates between the two groups (IVT + CAS: 10.6% vs. CAS alone: 10.9%; OR = 0.78; 95% CI 0.44–1.37), with no heterogeneity (I^2^ = 0%) (Fig. 1B).

### Functional independence

**Fig. 2:**
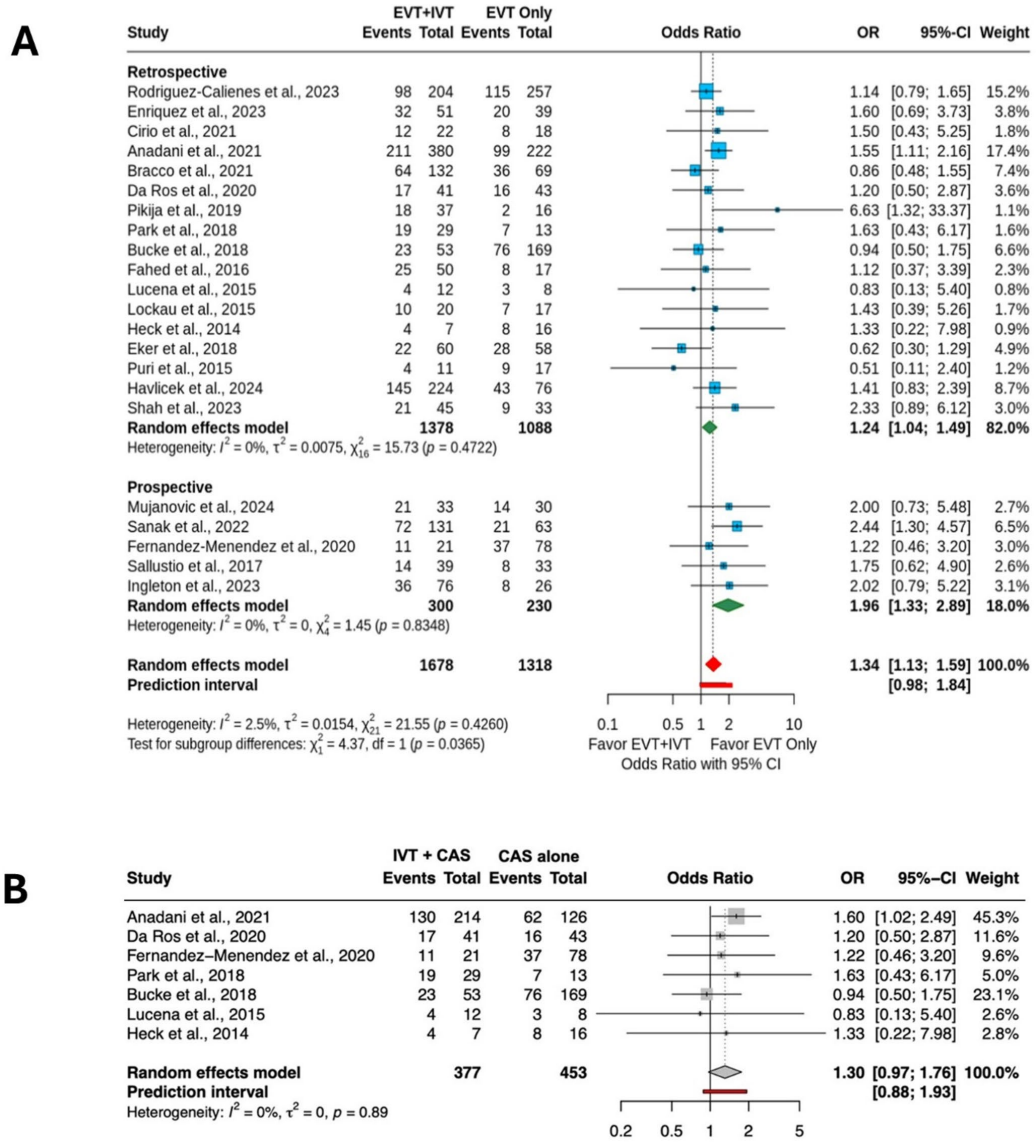
**A** Meta-analysis of functional independence in studies comparing intravenous thrombolysis plus endovascular thrombectomy (IVT + EVT) vs. endovascular thrombectomy (EVT) alone. **B** Sensitivity analysis of studies comparing IVT plus emergent carotid artery stenting (IVT + CAS) vs. CAS alone

Functional outcome data were available for 22 studies (2,996 patients). The IVT + EVT group had a 34% increase in the odds of 90-day functional independence (mRS score 0–2) compared with the EVT alone group (IVT + EVT: 52.6% vs. EVT alone: 44.1%; OR = 1.34; 95% CI 1.13–1.59) with low heterogeneity (I^2^ = 2.5%). The effect size was larger in prospective studies (OR = 1.96; 95% CI 1.33–2.89) than in retrospective studies (OR = 1.24; 95% CI 1.04–1.49) (Fig. 2A). Meta-regression showed no significant effect of baseline variables (age: *P* = 0.62, I² = 18.1%; NIHSS: *P* = 0.69, I² = 12.3%) on the association between IVT + EVT and functional independence.

Seven studies (830 patients) assessed functional outcomes in IVT + CAS vs. CAS alone. The odds of 90-day functional independence were similar between groups, with a trend favoring IVT + CAS (IVT + CAS: 55.1% vs. CAS alone: 46.1%; OR = 1.30; 95% CI 0.97–1.76), with low heterogeneity (I^2^ = 0%) (Fig. 2B).

### Additional outcomes

**Fig. 3:**
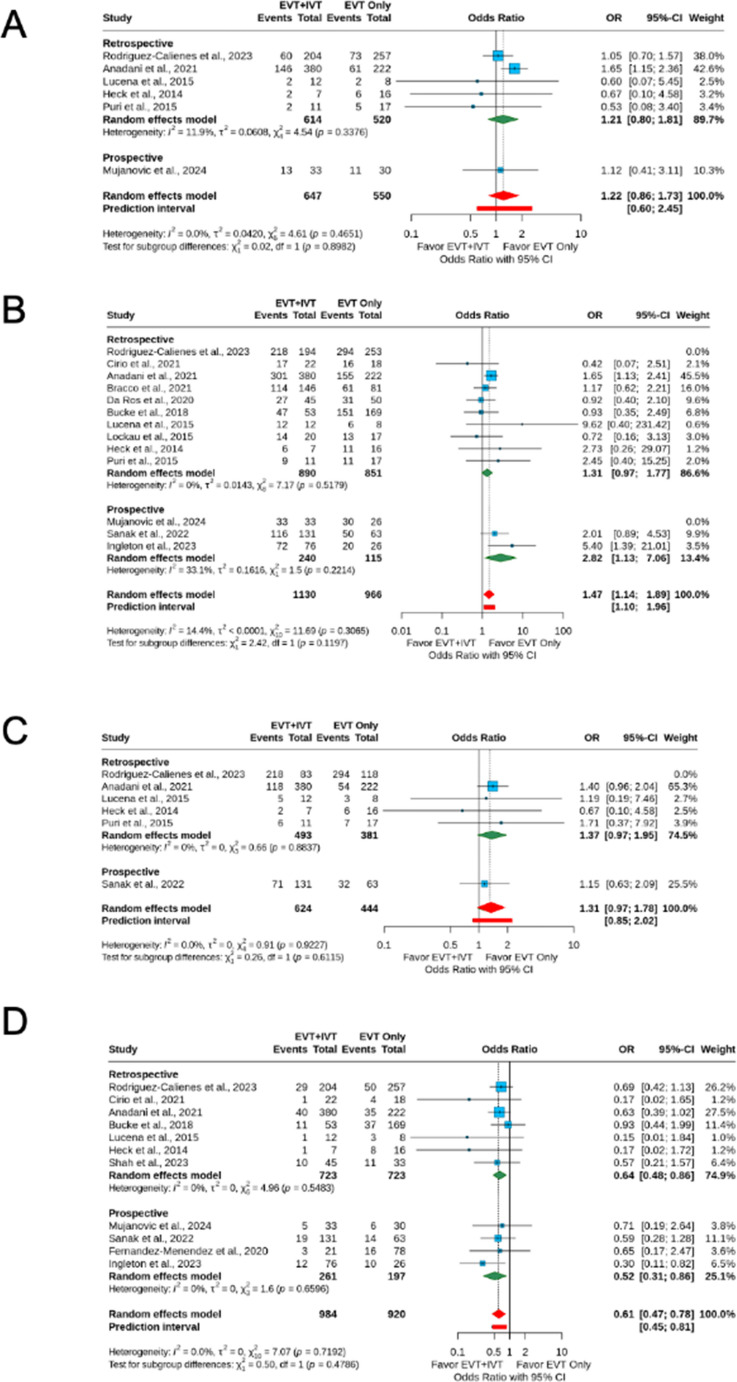
Forest plots for (**A**) excellent clinical outcome, (**B**) successful reperfusion (mTICI 2b-3), (**C**) complete reperfusion (mTICI 3), and (**D**) 90-day mortality

For the additional outcome of 90-day excellent clinical outcome (mRS score 0–1), data were available for 6 studies (1,197 patients). The odds for excellent clinical outcome were similar between groups (IVT + EVT: 34.7% vs. EVT alone: 28.7%; OR = 1.22; 95% CI 0.86–1.73) with no heterogeneity (I^2^ = 0%). For successful reperfusion (mTICI 2b-3), data were available for 13 studies (2,165 patients). The IVT + EVT group had 46% higher odds for successful reperfusion (IVT + EVT: 83.3% vs. EVT alone: 79.8%; OR = 1.47; 95% CI 1.14–1.89) with low heterogeneity (I^2^ = 14.4%). Rates for complete reperfusion (mTICI 3) were available for 6 studies (1,379). The odds for complete reperfusion were similar between groups (IVT + EVT: 37.5% vs. EVT alone: 35.4%; OR = 1.31; 95% CI 0.97–1.78) with no heterogeneity (I^2^ = 0%). The odds of mortality were overall 39% lower in the IVT + EVT group compared to the EVT alone group (IVT + EVT: 13.4% vs. EVT alone: 21.1%; OR = 0.61; 95%CI 0.47–0.78) with no heterogeneity (I^2^ = 0%). The funnel plots for additional outcomes are presented in Fig. 3.

## Discussion

This comprehensive meta-analysis of 24 studies comprising 3,294 patients represents the largest analysis to date on this topic. We found that in patients with TL, receiving IVT prior to EVT does not increase the risk of sICH compared to EVT alone. Notably, this finding remained consistent when studies including emergent CAS as the primary endovascular approach for carotid lesions were analyzed. Additionally, our findings indicate that IVT + EVT is associated with higher odds of achieving functional independence, improved reperfusion rates, and reduced 90-day mortality. By including a substantially larger sample size than previous meta-analyses and uniquely examining outcomes in studies where CAS was the primary approach for cervical lesions, our study provides a more robust and clinically relevant assessment to guide treatment strategies in TL management.

A major concern when combining IVT with EVT in the context of TL is the increased risk of hemorrhagic complications, particularly in patients requiring emergent CAS. This risk contributes to the considerable variation and uncertainty in determining the optimal endovascular approach [8]. Stent placement in the carotid artery requires additional antiplatelet therapy—whether single, dual, intravenous, or a combination— to prevent acute in-stent thrombosis and reduce the likelihood of extracranial ICA restenosis [41–43]. However, in this setting, the concurrent use of IVT and antiplatelet therapy, while essential for maintaining intracranial and cervical arterial patency, must be carefully weighed against the heightened risk of intracranial hemorrhage.

Despite these concerns, our study found that IVT before EVT did not increase the risk of sICH in patients with TL, even in the setting of emergent CAS. These findings suggest that IVT can be safely administered in this stroke population without significantly increasing the risk of hemorrhagic complications. However, if IVT is chosen as the initial treatment, careful management of bleeding risk factors and thoughtful approach to antiplatelet selection remain crucial. Given this, an important next step is to identify the safest and most effective antiplatelet regimen when IVT is administered prior to emergent CAS. A recent meta-analysis suggests that “high-intensity antiplatelet therapy”, including dual antiplatelet therapy or glycoprotein IIb/IIIa inhibitors, does not appear to significantly increase the risk of sICH in patients undergoing emergent CAS for TL [44]. Moreover, in line with our findings, this analysis reported that prior IVT did not influence the relationship between antiplatelet use and the risk of sICH [44]. 

A subgroup analysis from IRIS on TL found no significant difference in the 90-day mRS shift between the IVT + EVT and EVT-alone groups, with no significant interaction detected in this subgroup (*p*for interaction = 0.56) [9]. However, our study suggests that IVT + EVT offers greater benefits than EVT alone in achieving both functional independence (90-day mRS 0–2) and successful reperfusion in patients with TL. These findings align with a recent small meta-analysis of nine observational non-randomized studies, comprising 1,838 participants, which reported that IVT + EVT was associated with higher odds of favorable functional outcomes (90-day mRS 0–2) and improved recanalization rates compared to EVT alone [45]. 

The mechanism underlying these benefits may be explained by the role of IVT in enhancing intracranial flow restoration, which we previously reported to mediate 25% of the effect of emergent CAS on functional outcomes in TL [46]. We propose that IVT may further enhance intracranial flow restoration through a synergistic action on both extracranial and intracranial occlusions. At the extracranial level, IVT could reduce the in-situ thrombosis at the level of the stenosis or facilitate the microcatheter to cross the clot in cases of cervical carotid occlusion before stent deployment, thereby facilitating earlier delivery of fibrinolytic factors to the intracranial occlusion and enhancing fibrinolysis [47]. At the intracranial level, the thrombolytic agent itself may potentially soften the thrombus, thereby facilitating endovascular intervention to more effectively clear the clot.

### Limitations

Our systematic review and meta-analysis, which includes 24 studies with a total of 3,294 participants, has many strengths; however, certain limitations should be acknowledged. First, the inclusion of observational, non-randomized studies introduces a high risk of selection bias and residual confounding due to baseline imbalances and unmeasured variables between groups. In particular, the decision to administer IVT prior to EVT was not randomized, raising the possibility of allocation bias that may have influenced treatment effects despite multivariable adjustments in some studies. Our pooled population included patients treated in both the early and late time windows, which limited our ability to perform subgroup analyses by time window. However, existing evidence suggests that the treatment effect in patients with TL is generally comparable across both timeframes [48]. Second, a major limitation across all studies in our meta-analysis is the lack of detailed information on antiplatelet therapy regimens in the context of emergent CAS, reasons for withholding IVT, and data on stent patency at follow-up. Given that antiplatelet therapy is a critical confounder for the risk of sICH, especially in emergent CAS, its omission significantly limits our ability to interpret safety outcomes across studies. Furthermore, the variability in IVT protocols (thrombolytic agents, dosages) and definitions of sICH complicates the comparison of outcomes across studies and limits the generalizability of our findings. Third, our study did not address the potential benefits of administering IVT early at a primary stroke center (the drip and ship approach) versus direct transfer to a comprehensive stroke center (the mothership approach), leaving this important clinical question unanswered. Additionally, we were unable to evaluate the impact of the combined approach on door-to-groin puncture time due to the limited availability of this outcome stratified by treatment group in the included studies. Fourth, most studies in our analysis used alteplase as the thrombolytic agent, despite emerging evidence suggesting that tenecteplase may offer superior recanalization and clinical outcome [49]. Subgroup or sensitivity analyses based on thrombolytic type (alteplase vs. tenecteplase) were not conducted, which may limit the generalizability of our findings to populations treated with tenecteplase. Lastly, funnel plot asymmetry observed for functional independence and mortality outcomes suggests potential underreporting of negative results. However, this finding should be interpreted with caution due to the limited number of included studies.

Although these limitations should be considered when interpreting our findings, our results reflect real-world treatment practices and capture the same variability in treatment allocation observed across clinical settings. Notably, our study represents the largest meta-analysis comparing IVT + EVT to EVT alone for TL and is the only study to date to specifically compare IVT + CAS with CAS alone.

## Conclusion

In conclusion, the current meta-analysis provides supporting evidence indicating that the combination of IVT and EVT does not increase the risk of sICH compared to EVT alone in patients with TL, including those undergoing emergent CAS. Notably, IVT + EVT may offer potential benefits in achieving functional independence, successful reperfusion, and reducing mortality. Despite these encouraging findings, the conclusions are tempered by the reliance on observational, non-randomized data, which introduces the potential for confounding and selection bias. Variability in antiplatelet regimens, thrombolytic agents, and sICH definitions further complicates the interpretation of safety outcomes. Moreover, critical gaps remain in understanding the optimal timing and approach for IVT administration in TL, as well as the most effective antiplatelet strategies in the setting of emergent CAS. RCTs with standardized antiplatelet protocols are needed to validate these findings. Until such evidence becomes available, the decision to administer IVT prior to EVT in TL should remain individualized, balancing the potential for improved outcomes with the risks associated with hemorrhagic complications, particularly in the context of emergent CAS.

## Supplementary Information

Below is the link to the electronic supplementary material.

ESM 1 (DOCX 571 KB)

## Data Availability

No datasets were generated or analysed during the current study.
