# Supplementary material for: Intravenous thrombolysis before endovascular therapy for acute ischemic stroke due to tandem lesions: a systematic review and meta-analysis
Source: Neurosurg Rev. 2025 Sep 8;48(1):634. doi: 10.1007/s10143-025-03786-6 (PMC12417230; doi:10.1007/s10143-025-03786-6)
Supplement: Supplementary file 1 — (DOCX 571 KB) [file 10143_2025_3786_MOESM1_ESM.docx]

1. **Supplementary Figures**


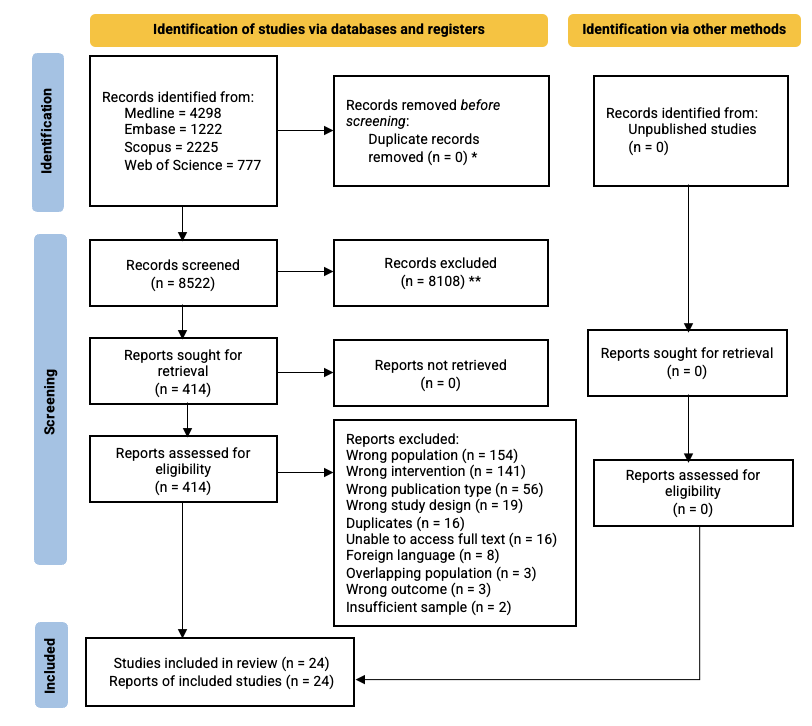


**[Supplementary Figure 1](#sf1)**. Flow chart of study selection.


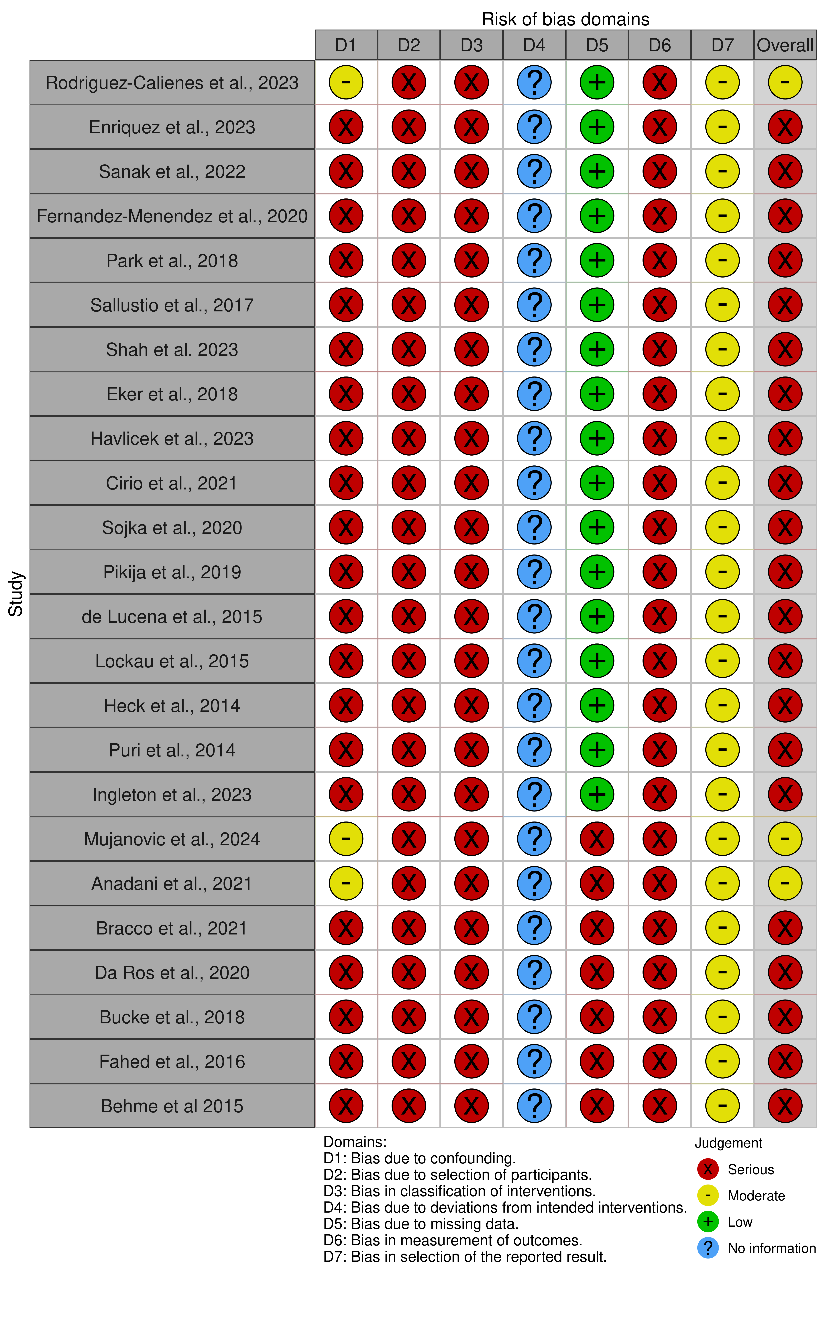


**[Supplementary Figure 2](#sf2)**. Risk of bias assessment.


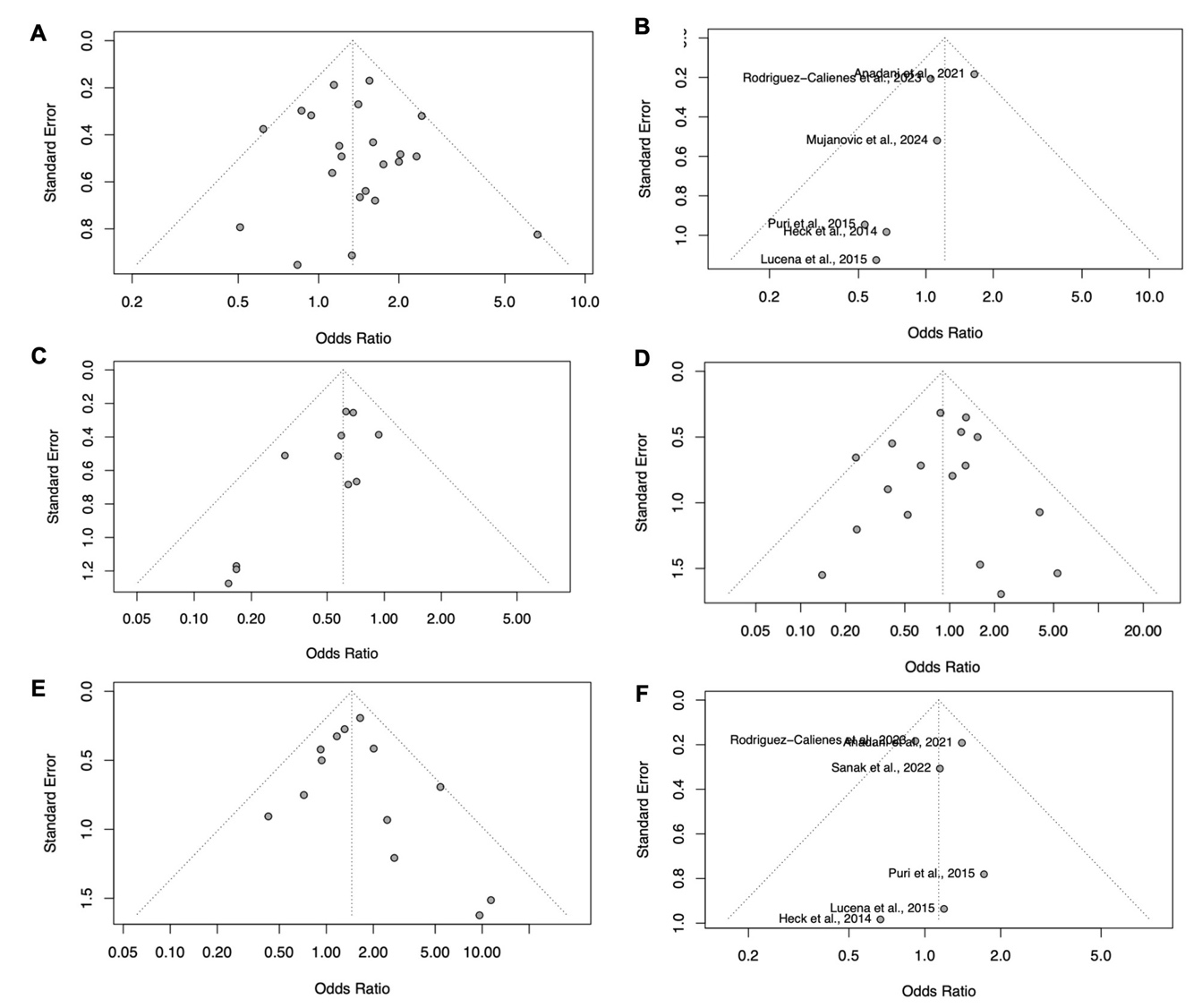


**[Supplementary Figure 3](#sf3)**. Funnel plots for (A) 90-day functional independence, (B) excellent clinical outcome, (C) 90-day mortality, (D) symptomatic intracranial hemorrhage, (E) mTICI 2b-3, and (F) mTICI 3.

1. **Supplementary** **Tables**

| **[Supplementary Table 1](#ST1).** Search strategy. | | |
| --- | --- | --- |
| **Database** | **Search strategy** | **Results** |
| Pubmed/ Medline | #1 ((tandem[ti]) and (occlusions[ti])) OR ((tandem[ti]) and (lesion*[ti])) OR ((occlusions[ti]) and (carotid[ti])) OR ( stroke[ti] OR “cerebral infarction”[ti] OR "carotid artery"[ti] )  #2 (((((( thromb*[ti] ) AND ( endovascular[ti] OR percutaneous[ti] OR catheter[ti] or intracranial[ti] or intervention[ti]))) OR (( endovascular[ti] and management[ti] ))) OR (( therapy[ti] and endovascular[ti] ) OR ( treatment[ti] and endovascular[ti] ))) OR (( embolectomy[ti] and mechanical[ti] ) OR ( thromb*[ti] and mechanical[ti] ) OR ( antithrombotic[ti] and treatment[ti] ) OR (Thromb*[ti] and treatment[ti]) or ((Thromb*[ti]) and (cirulation[ti])) or (Thromb*[ti] and intervention[ti]))) OR ((evt[ti]))  #3 ((((((intravenous[ti]) and (thrombolysis[ti]))) OR (((thromb*[ti] or intravenous[ti]) AND ( recombinant[ti] OR plasminogen[ti] OR tpa[ti] or alteplase[ti])))) OR ((Treatment[ti] and tPA[ti]) or (Administration[ti] and tPA[ti]))) OR ((ivt[ti]))) OR (((intravenous[ti]) and (alteplase[ti])))  #4 ((Thrombectomy[tiab] or thrombolysis[tiab]) AND (stroke[tiab]) and (carotid[tiab])) AND ((recanalization[tiab] or revascularization[tiab] or reperfusion[tiab] OR mortality[tiab] OR "mRS"[tiab] OR "modified Rankin"[tiab] OR predictor[tiab] OR aspects[tiab] OR nihss[tiab] OR hemorrhagic[tiab] OR "intracranial hemorrhage"[tiab] OR sich[tiab] OR tici[tiab] OR mtici[tiab] OR "collateral flow"[tiab] OR "clinical results"[tiab] OR "favorable clinical"[tiab] OR efficacy[tiab]))  #5 #2 OR #3  #6 #1 AND #5  #7 #4 OR #6 | 4537 |
| Scopus | #1 TITLE( tandem W/2 occlusions ) OR TITLE( tandem w/2 lesion*) OR TITLE( occlusions W/2 carotid ) OR TITLE( stroke OR "carotid artery" )  #2 ( TITLE( thromb* ) AND TITLE( endovascular OR percutaneous OR catheter or intracranial or intervention) ) OR TITLE( endovascular W/2 management ) OR TITLE( therapy W/2 endovascular ) OR TITLE( treatment W/3 endovascular ) OR TITLE( embolectomy W/3 mechanical ) OR TITLE ( thromb* W/2 mechanical ) OR TITLE( antithrombotic W/2 treatment ) OR TITLE(Thromb* w/2 treatment) or TITLE(Thromb* w/2 cirulation)  #3 (TITLE( intravenous W/2 thrombolysis ) OR TITLE( intravenous W/2 alteplase ) OR TITLE(Treatment w/2 tPA) or TITLE(Administration w/3 tPA) OR (TITLE( thromb*) AND TITLE( recombinant OR plasminogen* OR tpa or alteplase)))  #4 ABS ( reperfusion OR recanalization OR mortality OR "mrs" OR "modified rankin" OR predictor OR aspects OR nihss OR hemorrhagic OR scenario OR "intracranial hemorrhage" OR sich OR tici OR mtici OR "collateral flow" OR "clinical results" OR "favorable clinical" OR efficacy )  #5 #2 OR #3  #6 #4 AND #5  #7 #1 AND #6 | 4508 |
| Web Of Science | #1 TI=( tandem NEAR/1 occlusions ) OR TI=( tandem NEAR/2 lesion*) OR TI=( stroke OR "carotid artery" )  #2 (TI=( thromb* ) AND TI=( endovascular OR percutaneous OR catheter or intracranial or intervention) ) OR TI=( endovascular NEAR/2 management ) OR TI=( therapy NEAR/2 endovascular ) OR TI=( treatment NEAR/3 endovascular ) OR TI=( embolectomy NEAR/3 mechanical ) OR TI=( thromb* NEAR/2 mechanical ) OR TI=( antithrombotic NEAR/2 treatment ) OR TI=(Thromb* NEAR/2 treatment) or TI=(Thromb* NEAR/2 cirulation)  #3 (TI=( intravenous NEAR/2 thrombolysis ) OR TI=( intravenous NEAR/2 alteplase ) OR TI=(Treatment NEAR/4 tPA) or TI=(Administration NEAR/3 tPA) OR (TI=( thromb*) AND TI=( recombinant OR plasminogen* OR tpa or alteplase)))  #4 AB=( reperfusion OR recanalization OR mortality OR "mRS" OR "modified Rankin" OR predictor OR aspects OR nihss OR hemorrhagic OR scenario OR "intracranial hemorrhage" OR sich OR tici OR mtici OR "collateral flow" OR "clinical results" OR "favorable clinical" OR efficacy )  #5 #2 AND #3  #6 #1 AND #5  #7 #6 AND #4 | 3681 |
| Embase | #1 (((lesion* OR tandem) NEAR/2 tandem):ti) AND ([embase]/lim OR [embase classic]/lim OR [preprint]/lim)  #2 (stroke:ti OR 'carotid artery':ti) AND ([embase]/lim OR [embase classic]/lim OR [preprint]/lim)  #3 #2 or #1  #4 thromb*:ti AND ([embase]/lim OR [embase classic]/lim OR [preprint]/lim)  #5 (endovascular:ti OR percutaneous:ti OR catheter:ti OR intracranial:ti OR intervention:ti) AND ([embase]/lim OR [embase classic]/lim OR [preprint]/lim)  #6 #4 and #5  #7 (((treatment OR therapy OR management) NEAR/2 endovascular):ti) AND ([embase]/lim OR [embase classic]/lim OR [preprint]/lim)  #8 (((circulation OR treatment OR mechanical) NEAR/2 thromb*):ti) AND ([embase]/lim OR [embase classic]/lim OR [preprint]/lim)  #9 (((intravenous OR recombinant OR plasminogen* OR tpa OR alteplase) NEAR/2 thromb*):ti) AND ([embase]/lim OR [embase classic]/lim OR [preprint]/lim)  #10 #6 or #7 or #8 or #9  # 11 #3 and #10  #12 (reperfusion:ti OR recanalization:ti OR mortality:ti OR 'mrs':ti OR 'modified rankin':ti OR predictor:ti OR aspects:ti OR nihss:ti OR hemorrhagic:ti OR scenario:ti OR 'intracranial hemorrhage':ti OR sich:ti OR tici:ti OR mtici:ti OR 'collateral flow':ti OR 'clinical results':ti OR 'favorable clinical':ti OR efficacy:ti OR insights:ti) AND ([embase]/lim OR [embase classic]/lim OR [preprint]/lim)  #13 #11 and #12 | 2105 |

| **[Supplementary Table](#st2)****[2](#st2)**. Inclusion and exclusion criteria for study eligibility. | | |
| --- | --- | --- |
|  | **Inclusion criteria** | **Exclusion criteria** |
| **Population** | - Adult patients (≥18 years old) presenting with acute ischemic stroke secondary to tandem lesions. - Tandem lesions were defined according to each study definition. | - Children and adolescent patients (<18 years old). - Patients presenting with acute ischemic stroke secondary to any etiology other tandem lesions. |
| **Intervention** | IVT with a fibrinolytic agent (i.e., alteplase or tenecteplase) and one or more of the following techniques for intracranial thrombectomy:   - Stent retrievers - Aspiration catheters - Any combination of the aforementioned.   For the cervical endovascular approach, we considered:   - Angioplasty alone - Angioplasty and emergent carotid stenting | Delayed cervical endovascular treatment:   - Carotid artery stenting - Carotid endarterectomy - Trans-carotid artery revascularization |
| **Comparator** | Endovascular therapy without the use of IVT | |
| **Follow-up time** | We will include randomized and nonrandomized studies with a minimum follow-up time of 3 months. | |
| IVT: Intravenous thrombolysis. | | |

| **Supplementary Table 3**. Egger’s test for prioritized outcomes. | |
| --- | --- |
| **Outcome** | **p-value** |
| Complete repefusion | Not valid |
| Succesful reperfusion | 0.839 |
| Symptomatic intracranial hemorrhage | 0.649 |
| 90-day mortality | 0.0245 |
| Excellent clincal outcome | Not valid |
| Functional independence | 0.647 |
| Not valid coefficients were not calculated due to the limited number of studies for these outcomes. | |
